# Potent Anti-Cancer Effect of 3′-Hydroxypterostilbene in Human Colon Xenograft Tumors

**DOI:** 10.1371/journal.pone.0111814

**Published:** 2014-11-12

**Authors:** Tzu-Chun Cheng, Ching-Shu Lai, Min-Ching Chung, Nagabhushanam Kalyanam, Muhammed Majeed, Chi-Tang Ho, Yuan-Soon Ho, Min-Hsiung Pan

**Affiliations:** 1 Graduate Institute of Medical Sciences, College of Medicine, Taipei Medical University, Taipei, Taiwan; 2 Institute of Food Science and Technology, National Taiwan University, Taipei, Taiwan; 3 Sabinsa Corporation, East Windsor, New Jersey, United States of America; 4 Department of Food Science, Rutgers University, New Brunswick, New Jersey, United States of America; 5 Department of Laboratory Medicine, Taipei Medical University Hospital, Taipei, Taiwan; 6 School of Medical Laboratory Science and Biotechnology, College of Medical Science and Technology, Taipei Medical University, Taipei, Taiwan; 7 Comprehensive Cancer Center, Taipei Medical University, Taipei, Taiwan; 8 Department of Medical Research, China Medical University Hospital, China Medical University, Taichung, Taiwan; National Cheng Kung University, Taiwan

## Abstract

Here we report that 3′-hydroxypterostilbene (HPSB), a natural pterostilbene analogue, was more potent than pterostilbene against the growth of human cancer cells (COLO 205, HCT-116, and HT-29) with measured IC_50_ values of 9.0, 40.2, and 70.9 µM, respectively. We found that HPSB effectively inhibited the growth of human colon cancer cells by inducing apoptosis and autophagy. Autophagy occurred at an early stage and was observed through the formation of acidic vesicular organelles and microtubule-associated protein 1 light chain 3-II production. At the molecular levels, the results from western blot analysis showed that HPSB significantly down-regulated phosphatidylinositol 3-kinase (PI3K)/Akt and mitogen-activated protein kinases (MAPKs) signalings including decreased the phosphorylation of mammalian target of rapamycin (mTOR). Significant therapeutic effects were demonstrated *in vivo* by treating nude mice bearing COLO 205 tumor xenografts with HPSB (10 mg/kg *i.p.*). These inhibitory effects were accompanied by mechanistic down-regulation of the protein levels of cyclooxygenase-2 (COX-2), matrix metallopeptidase-9 (MMP-9), vascular endothelial growth factor (VEGF), and cyclin D1, as well as by the induction of apoptosis in colon tumors. Our findings suggest that HPSB could serve as a novel promising agent for colon cancer treatment.

## Introduction

Epidemiological studies provide convincing evidence that dietary factors can modify the processes of carcinogenesis, including initiation, promotion, and progression of several types of human cancer [1]. Cancer chemoprevention is the use of pharmacological or natural agents to inhibit the development of invasive cancer or reverse the process of carcinogenesis. It could be the most direct process to reduce morbidity and mortality from cancerous disease [2]–[4]. A large number of chemopreventive and chemotherapeutic agents, from natural products, have been used as a promising strategy to fight against cancer by inducing apoptosis in malignant cells [5], [6].

**Figure 1:**
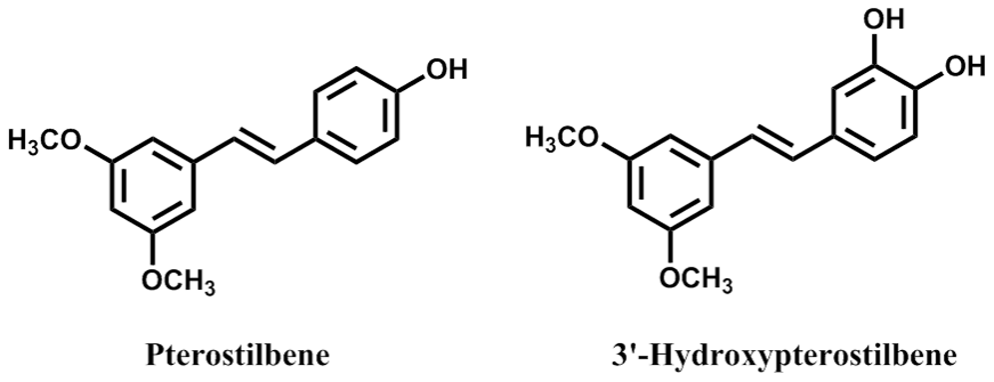
Chemical structure of pterostilbene and 3′-hydroxypterostilbene.

Pterostilbene (*trans*-3,5-dimethoxy-4′-hydroxystilbene), a dimethylether analogue of resveratrol, is found to be as effective as resveratrol in preventing carcinogen-induced preneoplastic lesions in a mouse mammary culture model and inhibits metastatic growth of melanoma cells to the liver [7], [8]. We and others have shown that pterostilbene exhibits pleiotropic pharmacological effects including anti-inflammatory, antioxidant, anti-proliferative, anti-cancer, and pain-relieving activities in cell culture and animal studies [9]–[14]. Recently, 3′-hydroxypterostilbene (**Fig. 1**), a new natural pterostilbene analogue has been isolated from *Sphaerophysa salsula*, is markedly more active than pterostilbene in inducing apoptosis in sensitive and resistant leukemia cells [15], [16]. However, the *in vivo* antitumor effect of HPSB remains unclear.

Autophagy, also known as type II programmed cell death, is characterized by the formation of a double-membrane or isolation membrane which derived from a part of the endoplasmic reticulum (ER) [17] or from the cytolplasmic lipid pool [18]. The double-membrane forms autophagosome by sequester portions of the cytoplasm and intracellular organelles [11], [12], [19], [20].

Autophagy is a response of eukaryotic cells to various microenvironment stresses, including starvation, pathogen infestation and chemotherapy. Moreover, there are also several reports has shown that autophagy induction appears to facilitate successful therapy-induced killing of tumor cells [21], and pro-autophagic drugs such as temozolomide are promising candidates for selective killing of apoptosis-resistant glioblastomas [22].

The initiating signal for autophagy formation is poorly understood, whereas it has been established that several molecules and signaling pathways are implicated in regulating autophagy, such as PI3K-AKT-mTOR (phosphatidylinositol 3-kinase/protein kinase B/mammalian target of rapamycin), MAPKs, Raf-1-MEK1/2-ERK1/2 and PTEN (phosphatase and tensin homologue) pathways [10], [23]. PTEN and AKT are upstream regulators of the mTOR pathway which act as an inducer or inhibitor of autophage, respectively. AKT inhibits autophage by regulating downstream molecular 4E-BP1 (4 elongation-bind protein 1), p70S6K that result in promoting mRNA translation.

In this current study, we first examined the antiproliferative effects of pterostilbene and its natural 3′-hydroxy derivative on human colon cancer cells. Our results clearly demonstrated that HPSB was more potent than pterostilbene in inducing apoptosis in a dose-dependent manner in COLO 205 cells.

We further evaluated the molecular mechanisms of apoptotic and autophagic effects induced by HPSB. To elucidate the anticancer mechanism of HPSB, we investigated the signaling pathways related to HPSB-induced autophagy in human COLO 205 colon cancer cells. *In vivo* therapeutic efficacy was further examined by treating nude mice bearing COLO 205 tumor xenografts with 10 mg/kg *ip* HPSB. This study provides novel evidence that the hydroxyl derivative of pterostilbene could serve as a new and promising agent against human colon cancer.

## Materials and Methods

### 2.1 Reagents

Propidium iodide (PI), acridine orange (AO) and chloroquine (CQ) was purchased from Sigma Chemical (St. Louis, MO, USA). 2′,7′-Dichlorodihydrofluorescein diacetate (DCFH-DA) and 3,3′-dihexyloxacarbocyanine iodide (DiOC6) were purchased from Molecular Probes (Eugene, OR, USA). The PARP, DFF-45, LC-3 I/II, p-mTOR (Ser^2448^), mTOR, p-P70S6K (Thr^398^), p-P70S6K (Ser^371^), p-Akt (Ser473), Akt, PI3K, p-ERK1/2 (Thr202/Tyr204), p-JNK1/2 (Thr183/Tyr185), JNK1/2, p-p38 (Thr180/Tyr182), p38 and MMP-9 antibodies were purchased from Cell Signaling Technology (Beverly, MA). The p-PI3K p85α (Tyr508), ERK1/2, VEGF and cyclin D1 antibodies were purchased from Santa Cruz Biotechnology (Santa Cruz, CA). The caspase 3, 8 and 9 antibodies were purchased from Imgenex (San Diego, CA, USA). The COX-2 antibody was purchased from Transduction Laboratories (BD Biosciences, Lexington, KY). The β-actin antibody was purchased from Sigma Chemical Co. (St. Louis, MO). Pterostilbene and HPSB were obtained from Sabinsa Corp. (East Windsor, NJ). The purity of pterostilbene and HPSB was determined by HPLC as higher than 99.2%.

### 2.2 Cell culture

The human colon cancer cell lines COLO 205, HCT-116 and HT-29 were purchased from the American Type Culture Collection (Rockville, MD). Cell lines were grown in RPMI-1640 supplemented with 10% heat-inactivated fetal bovine serum (GIBCO BRL, Grand Island, NY), 100 units/mL of penicillin, 100 µg/mL of streptomycin), 2 mM L-glutamine (GIBCO BRL, Grand Island, NY), and were kept at 37°C in a humidified 5% CO_2_ incubator. Pterostilbene and HPSB were dissolved in dimethylsulfoxide (DMSO, as final concentration of 0.05%). Cells were treated with 0.05% DMSO as vehicle control.

### 2.3 Cytotoxicity assay

Cell viability was assayed by 3-(4,5-dimethylthiazol-2-yl)-2,5-diphenyl tetrazolium bromide (MTT). Briefly, COLO 205, HCT-116 and HT-29 cells were plated at a density of 2×10^5^ cells/mL into 96-well plates. After overnight growth, cells were treated with a series of concentrations of pterostilbene or 3′-hydroxypterostilbene for 24 h. The final concentrations of DMSO in the culture medium were <0.05%. At the end of treatment, 0.2% MTT was added and cells were incubated for a further 4 h. Cell viability was determined by scanning with an ELISA reader with a 570-nm filter.

### 2.4 Flow cytometry analysis of sub-G1 cell population, mitochondrial membrane potential and ROS production

For sub-G1 cell population analysis, COLO 205 cells (2×10^5^ cells/mL) were cultured in 12 well and treatment with various concentrations of pterostilbene or 3′-hydroxypterostilbene for 24 h. The cells were then harvested, washed with PBS, resuspended in 200 µL of PBS, and fixed in 800 µL of iced 100% ethanol at -20°C. After being left to stand overnight, the cell pellets were collected by centrifugation, resuspended in 1 mL of hypotonic buffer (0.5% Triton X-100 in PBS and 0.5 µg/mL RNase) with PI (50 µg/mL) and incubated at 37°C in the dark for 30 min. Fluorescence emitted from the PI-DNA complex was quantitated after excitation of the fluorescent dye by FACScan cytometry (Becton Dickinson, San Jose, CA). In the autophagy inhibitor study, cells were pre-treated 25 µM CQ for 1 h, followed by incubation with pterostilbene or HPSB.

For mitochondrial membrane potential and ROS production, cell were treated as described above for 15 min, and then DiOC6 (40 nM) or DCFH-DA (20 µM) was added to the medium for a further 30 min at 37°C. After washing with PBS, the fluorescence intensity was determined by FACScan cytometry.

### 2.5 Detection of autophagy

Autophagy induction was detected by AO staining. After 24 h treatment, COLO205 cells were washed with PBS, suspended in PBS and stained with 1 µg/mL AO of 20 min. Photographs were obtained with a fluorescence microscope (Axioscop, Carl Zeiss, Thomwood, NY) equipped with a mercury 100-W lamp, 490-nm band-pass blue excitation filters, a 500-nm dichroic mirror and a 515-nm long-pass barrier filter. For quantification of AVOs by flow cytometry, cells were treated as described and stained with AO for 15 min and were analyzed by FACScan laser flow cytometer and CellQuest software.

### 2.6 Western Blotting

The total proteins of COLO 205 cells were extracted via addition of gold lysis buffer (50 mM Tris-HCl, pH 7.4; 1 mM NaF; 150 mM NaCl; 1 mM EGTA; 1 mM phenylmethanesulfonyl fluoride; 1% NP-40; and 10 µg/mL leupeptin) to the cell pellets on ice for 30 min, followed by centrifugation at 10,000×g for 30 min at 4°C. The total proteins were measured by Bio-Rad Protein Assay (Bio-Rad Laboratories, Munich, Germany). The samples (50 µg of protein) were mixed with 5× sample buffer containing 0.3 M Tris-HCl (pH 6.8), 25% 2-mercaptoethanol, 12% sodium dodecyl sulfate (SDS), 25 mM EDTA, 20% glycerol, and 0.1% bromophenol blue. The mixtures were boiled at 100°C for 5 min and were subjected to 10% SDS-polyacrylamide minigels at a constant current of 20 mA. Electrophoresis was then carried out on SDS-polyacrylamide gels. Proteins on the gel were electrotransferred onto an immobile membrane (PVDF; Millipore Corp., Bedford, MA) with transfer buffer composed of 25 mM Tris-HCl (pH8.9), 192 mM glycine, and 20% methanol. The membranes were blocked with blocking solution containing 20 mM Tris-HCl, and then immunoblotted with different primary antibodies and β-actin. The blots were rinsed three times with PBST buffer (0.2% Tween 20 in 1× PBS buffer) for 10 min each. Then blots were incubated with 1∶5000 dilution of the horseradish peroxidase (HRP)-conjugated secondary antibody (Zymed Laboratories, San Francisco, CA) and then washed again three times with PBST buffer. The transferred proteins were visualized with an enhanced chemiluminescence detection kit (ECL; Amersham Pharmacia Biotech, Buckinghamshire, UK). The densities of the bands were quantified with a computer densitometer (AlphaImagerTM 2200 System Alpha densitometer. Innotech Corporation, San Leandro, CA).

### 2.7 Activity of caspases

The caspase 3, 8 and 9 activity in protein extractions of COLO 205 cells was determined by a fluorogenic assay (Promega's CaspACE Assay System, Madison, WI). Briefly, 50 µg of total protein, as determined by the Bio-Rad protein assay kit (Bio-Rad Laboratories), was incubated with 50 µM substrate Ac-Asp-Glu-Val-Asp-methylcoumaryl-7-amine (caspase-3 specific substrate), Ac-Ile-Glu-Thr-Asp-AMC (Ac-IETD-AMC) (caspase-8 specific substrate), or Ac-Leu-Glu-His- Asp-AMC (Ac-LEHD-AMC) (caspase-9 specific substrate) at 30°C for 1 h. The release of methylcoumaryl-7-amine was measured by excitation at 360 nm and emission at 460 nm using a fluorescence spectrophotometer (ECLIPSE, Varian, Palo Alto, CA).

### 2.8 COLO 205 xenograft model

Male Balb/c nude mice at 3–4 weeks old (weighing 16–18 g) were obtained from the BioLASCO Experimental Animal Center (BioLASCO, Taipei, Taiwan). All animals were maintained in pathogen-free sterile isolators and in a controlled atmosphere (25±1 °C at 50% relative humidity) and with a 12 h light–12 h dark cycle according to institutional guidelines. Animals were fed with standard AIN-76 diet and all food, water, caging, and bedding were sterilized prior to use. Animals had free access to food and water at all times. Food cups were replenished with fresh diet every day. All animal experimental protocol used in this study was approved by Institutional Animal Care and Use Committee of the National Kaohsiung Marine University (IACUC, NKMU, #099-AAA9-02, validity dates: 08/01/2009-07/31/2012). After 1 week of acclimation, colon cancer COLO 205 cells (5×10^6^) in 0.2 mL PBS were injected subcutaneously between the scapulae of each nude mouse. After transplantation, tumor size was measured using calipers, and the tumor volume was estimated according to the following formula: tumor volume (mm^3^) = L×W2/2, where L is the length and W is the width. Once tumors reached a mean size of 100–200 mm^3^, mice were randomly divided into three groups (6 animals/group). Mice were *i.p*. injection with pterostilbene or 3′-hydroxypterostilbene (10 mg/kg/d, respectively) for 15 days, while control animals were received injection of corn oil. The diet intake and body weight of each animal was monitored every day. The tumor volume was assessed and recorded every 5 days using caliper measurements. After 15 days, the mice were sacrificed by CO_2_ asphyxiation and the liver, kidneys, spleen and solid tumors were excised immediately and weighed. Average tumor volume and tumor weight of each group were represent the mean ±standard deviation (SD). The tumor tissues were cut into several portion for western bolt analysis or stored at −80°C.

### 2.9 Statistical analysis

Data were presented as means ±SE for the indicated number of independently performed experiments. Comparisons of statistical significance between groups were made by one way Student's t-test or one-way analysis of variance (ANOVA). A *P*-value <0.05 was considered statistically significant.

## Results

### 3.1 Inhibition of cell proliferation in HPSB-treated human colon cancer cells

**Figure 2:**
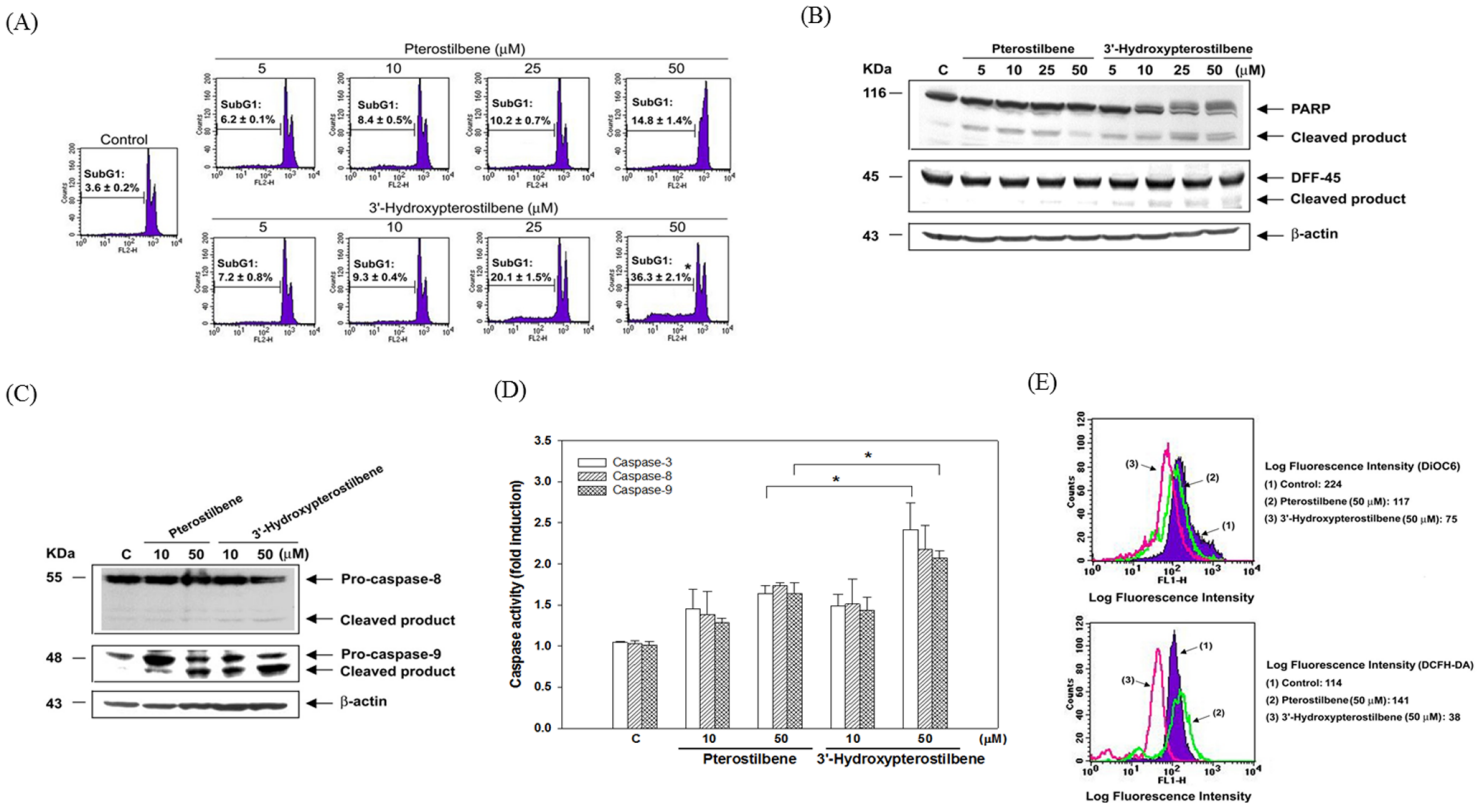
Pterostilbene and 3′-hydroxypterostilbene induced apoptosis in COLO205 cancer cells. Cells were treated with various concentrations (5, 10, 25 and 50 µM) of pterostilbene or 3′-hydroxypterostilbene for 24 h. (A) Determination of sub-G1 cells in COLO 205 cells by flow cytometry after PI staining as described in the Materials and Methods. (B, C) After treatment, total cell lysates were prepared from COLO 205 cells and the cleavage of PARP, DFF-45, pro-caspase 8 and pro-caspase 9 were analyzed by Western blotting. (D) Kinetics of caspase activation in COLO 205 cells. Cells were treated with 25 and 50 µM of pterostilbene or 3′-hydroxypterostilbene for 24 h. Caspase activities were analyzed as described in the Materials and Methods. (E) Cells were treated with 50 µM of pterostilbene or 3′-hydroxypterostilbene for 15 min. Mitochondrial membrane potential and ROS production were stained with DiOC6 (40 nM) and DCFH-DA (20 µM) and measured by flow cytometry. The values are expressed as means ±SE of triplicate tests. *P<0.05 indicates statistically significant difference from the pterostilbene-treated group.

We first compared the effects of pterostilbene and HPSB (**Figure 1**) on the growth of human colon cancer cells using the trypan blue exclusion assay as previously described. As shown in **Figure 2**, HPSB decreased cell growth in cultured human colon cancer cells (COLO 205, HCT-116, and HT-29) in a dose-dependent manner, with IC_50_ values of 9.0, 40.2, and 70.9 µM, respectively (Table 1). The cell-growth inhibitory effect on COLO 205 and HCT-116 cells (p53 wild-type) was sensitive to HPSB as compared to HT-29 (p53 mutant). These results suggest that p53 could be a key regulator in COLO 205 cells. Compared to pterostilbene, HPSB was a stronger inhibitor of COLO 205 cell growth. As a result, we further examined the cytotoxic effects of HPSB in COLO 205 cells.

**Table 1:** Effect of pterostilbene and 3′-hydroxypterostilbene on the growth of colon cancer cells^a^.

	Compound IC_50_ (µM)	
Cell line	Pterostilbene	3′-hydroxypterostilbene
COLO205	33.4±0.2	9.0±0.2
HCT-116	47.1±0.6	40.2±0.6
HT-29	80.6±3.3	70.9±1.0

a Cells were treated with various concentrations of selected compounds for 24 h. Cell viability then was determined by the MTT assay as described. Each experiment was independently performed three times and expressed as mean ±SD.

### 3.2 HPSB induced apoptosis in human colorectal carcinoma cells

Flow cytometry was used to investigate the induction of a sub-G1 cell population, a hallmark of apoptosis. To investigate whether the cytotoxic effects of HPSB observed in COLO 205 cells were due to apoptotic cell death, cells were treated with pterostilbene and HPSB (5–100 µM) for 24 h and DNA content of COLO 205 cells were performed by flow cytometry (**Figure 2A**). As shown in **Figure 2A**, the percentages of apoptotic COLO 205 cells was 6.2, 8.4, 10.2, 14.8, and 7.2 and 9.3, 20.1, and 36.3% after incubation with 5, 10, 25, and 50 µM pterostilbene and HPSB, respectively. **Figure 2B** compared with pterostilbene, HPSB markedly cause the degradation of 116 kDa PARP into 85 kDa fragments and induce DFF-45 protein degradation. These protein cleavages were associated with the activation of caspase-3. As shown in **Figure 2C**, HPSB induced a dramatic increase in caspase-9 cleavage than pterostilbene. However, only less effect on caspase-8 activity in HPSB treated cells. To monitor the enzymatic activity of caspase-3, -8, and -9, caspase activity was measured following treatment of COLO 205 cells with 10 and 50 µM HPSB. As shown in **Figure 2D**, HPSB induced a dramatic increase in caspase-3 activity of approximately 2.4-fold after 24 h of treatment. It has recently become clear that apoptosis involves a disruption of mitochondrial membrane integrity that is decisive for the cell-death process. We therefore evaluated the effects of HPSB on the mitochondrial trans-membrane potential (ΔΨ_m_). Results of measuring fluorescence intensity in COLO 205 cells exposed to pterostilbene and HPSB compared to untreated control cells are summarized in **Fig. 2E**. The DiOC6(3) fluorescence intensity shifted to the left from 224 to 117 and 75 in pterostilbene and HPSB-induced apoptotic COLO 205cells, respectively. These results confirmed that HPSB caused a decrease in the mitochondrial trans-membrane potential in COLO 205 cells. The role of ROS in the induction of apoptosis is well recognized. Interestingly, HPSB markedly decreased the mean DCFH-DA fluorescence intensity from 114 to 38, whereas pterostilbene increased DCFH-DA fluorescence intensity from 114 to 141 at 15 min. The imbalance of ROS concentrations could plays an important role as an early mediator in HPSB-induced apoptosis.

### 3.3 HPSB showed stronger inducing effects on autophagy than pterostilbene in COLO 205 cells

**Figure 3:**
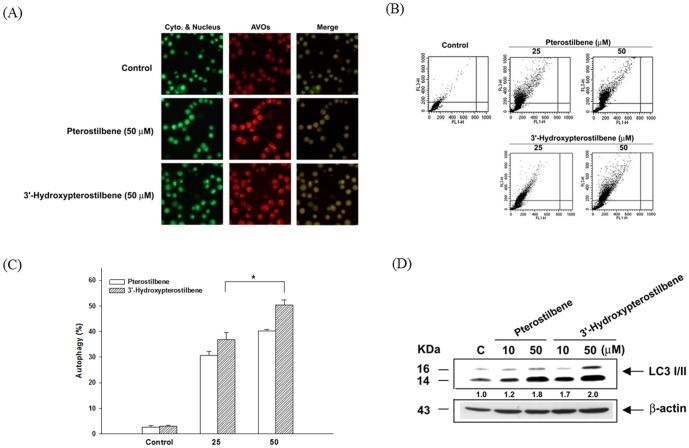
Autophagy induction by pterostilbene and 3′-hydroxypterostilbene in COLO 205 cancer cells. Cells were treated with 50 µM pterostilbene or 3′-hydroxypterostilbene for 24 h and stained with acridine orange. (A) Green and red fluorescence in acridine orange-stained cells were observed under fluorescence microscope. (B, C) Detection and quantification of autophagy in COLO 205 cells. Cells were treated with 25 and 50 µM of pterostilbene or 3′-hydroxypterostilbene for 24 h and stained with acridine orange. The measurement of green and red fluorescence in acridine orange-stained cells was performed using flow cytometry. (D) Cell lysates were prepared after 24 h treatment and the protein expression of LC3 I/II were analyzed by Western blotting. Data were presented as mean ±SD of triplicate experiments. *P<0.05 indicates statistically significant difference from the pterostilbene-treated group.

Accumulating evidence clearly indicates that pterostilbene has anti-tumorigenesis ability through the induction of apoptosis and autophagy. We next assessed whether pterostilbene and HPSB also induced autophagy in COLO 205 cells. As shown in **Figure 3A**, HPSB treatment resulted in marked appearance of AVO than pterostilbene when cells were stained with acridine orange after 24 h treatment. To quantify the incidence of HPSB-induced autophagy, cells with AVOs showed enhanced red fluorescence analyzed by flow that significantly increased after treatment with HPSB in a dose-dependent manner (**Figure 3B and 3C**). To confirm the occurrence of autophagy induced by pterostilbene and HPSB, we examined the process of LC3I/II, hallmarks of autophagy, using immunoblot to detect cell-extracted lysates from COLO 205 cells. As shown in **Figure 3D**, HPSB more stronger increased the amounts of LC3B I/II proteins than pterostilbene.

### 3.4 HPSB inhibited the mTOR/p70S6K, PI3K/Akt and MAPKs signaling pathways in COLO 205 cells

**Figure 4:**
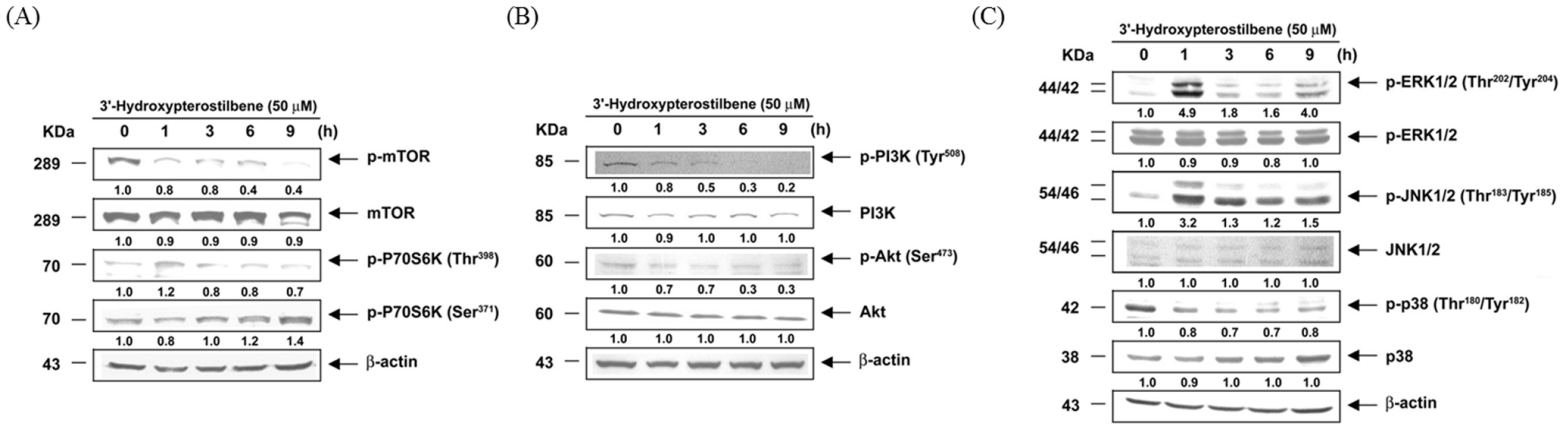
3′-Hydroxypterostilbene down-regulated mTOR, PI3K/Akt and MAPKs signaling in COLO 205 cancer cells. COLO 205 cells were treated with 50 µM 3′-hydroxypterostilbene at different times. Cell lyates were prepared and the protein levels of (A) p-mTOR, p-P70S6K, (B) p-PI3K, p-Akt and (C) p-ERK1/2, p-JNK1/2, p-p38 were analyzed by Western blotting analysis. All analyses were representative of at least three independent experiments. The values under each lane indicate relative density of the band normalized to β-actin using a densitometer.

To further understand the molecular mechanisms of HPSB induced autophagy in COLO 205 cells, we examined the phosphorylation of mTOR/p70S6K, PI3K/Akt, and MPAKs in HPSB treated cells. The results showed that phosphorylation of mTOR and p70S6K (Thr389) was markedly decreased in cells treated with HPSB (Figure 4A). Accumulating evidence supports that PI3K/Akt and the MAPKs pathways are involved in regulating autophagy, however, the JNK1/2 in this pathway remains to be clarified. Therefore, we investigated how these signaling pathways functioned in inducing autophagy by HPSB in COLO 205 cells. As shown in **Figure 4B and 4C**, the phosphorylation of PI3K, Akt, and p38 MAPK decreased in cells treated with HPSB in a time dependent manner. On the contrary, treatment with HPSB increased the phosphorylated ERK1/2 and JNK1/2 effectively for 1 h then gradually decreased the phosphorylation of ERK1/2 and JNK1/2 from 3 h to 9 h compared with total ERK1/2 and JNK1/2 protein in COLO 205 cells. Taken together, these results indicate that HPSB inhibits the PI3K/Akt/mTOR/p70S6K, and p38MAPK pathways and activates the ERK1/2, JNK1/2 MAPK pathways and suggests that these changes mediate HPSB-induced autophagy in COLO 205 cells.

**Figure 5:**
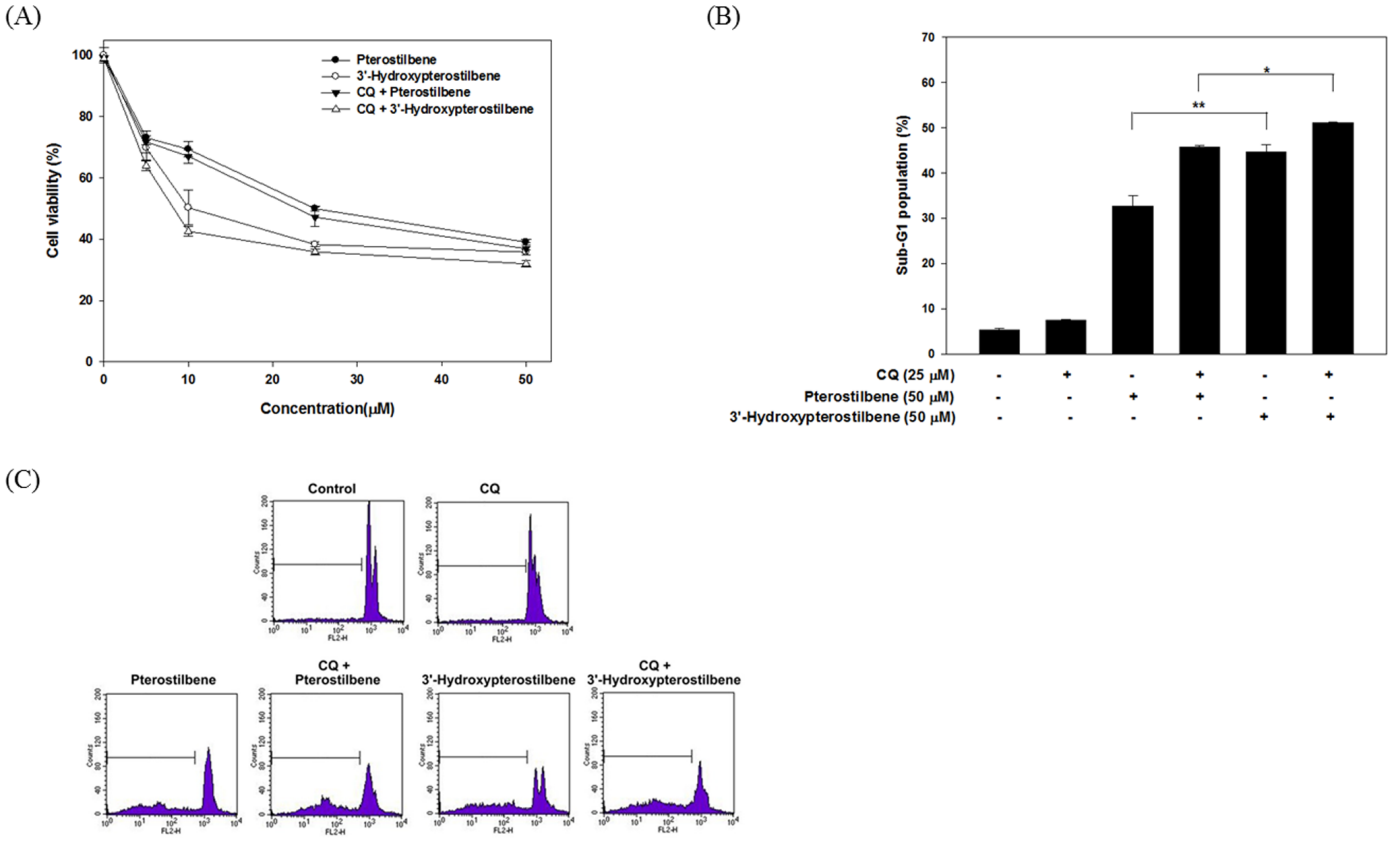
Autophagy inhibitor sensitized pterostilbene and 3′-hydroxypterostilbene-induced apoptosis in COLO 205 cancer cells. Cells were pretreated with 25 µM CQ for 1 h before treatment with 50 µM of pterostilbene or 3′-hydroxypterostilbene for 24 h. (A) Cell viability was determined by MTT assay. (B, C) Sub-G1 cell population (%) was analyzed and quantification after PI staining followed by flow cytometry. Data were presented as mean ±SD of triplicate experiments. ^*^ *P*<0.05 and ^**^ *P*<0.01 indicates statistically significant difference from the pterostilbene-treated group.

In addition, we used chloroquine (CQ), an inhibitor of autophagy, to determine whether inhibition of autophagy suppressed HPSB-induced cytotoxicity. As shown in **Figure 5**, the results indicated that treatment with CQ revealed significant increase cytotoxicity (**Fig. 5A**) by enhanced HPSB-triggered apoptosis (**Fig. 5B and 5C**). These results corroborate with the observation that HPSB treatment induces autophagic cell death in COLO 205 cells.

### 3.5 HPSB strongly inhibited tumor growth *in vivo*

**Figure 6:**
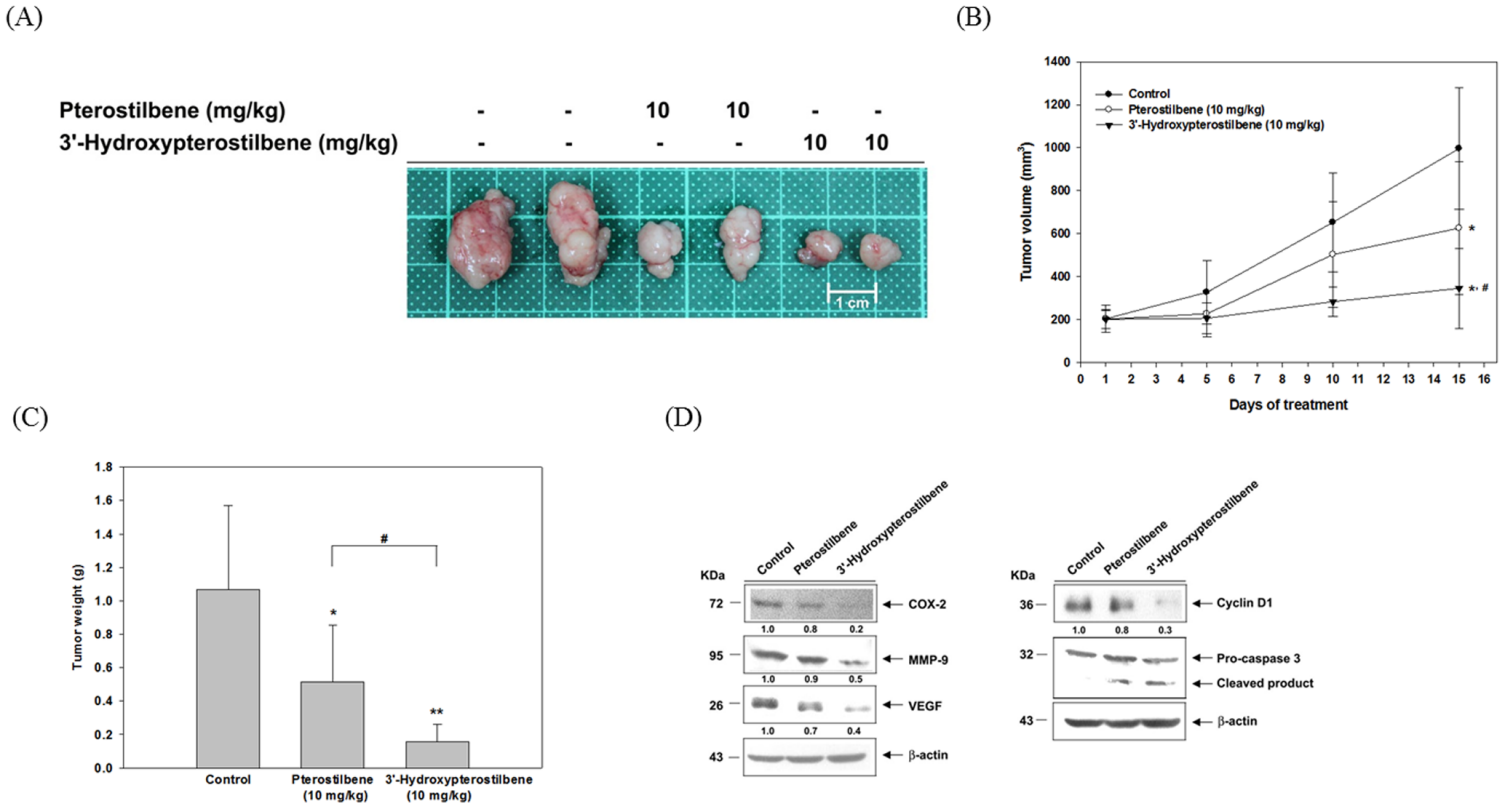
Pterostilbene and 3′-hydroxypterostilbene reduced the growth of COLO 205 xenografts in nude mice. Experimental treatment protocol as described in Materials and Methods. Mice bearing COLO 205 xenografts were *i.p.* injection with pterostilbene or 3′-hydroxypterostilbene for 15 days where control group was received corn oil. (A) Photograph of xenograft tumors developed in each group is shown at the end of day15. (B) Average tumor volumes were recorded during the treatment and (C) average tumor weights were measured at the end of experiment. Six samples were analyzed in each group, and values represent the mean ±SD. ^*^ *P*<0.05 and ^**^ *P*<0.01, compared with control group. ^#^ *P*<0.05, compared with pterostilbene-treated group. (D) Total proteins of xenograft tumors in each group were extracted for western blot analysis. COX-2, MMP-9, VEGF, PCNA, cyclin D1 protein expression and cleaved caspase-3 were detected by using specific antibodies. Similar results were obtained in three independent experiments.

We further examined the therapeutic efficacy of HPSB *in vivo* by treating nude mice bearing human colorectal carcinoma COLO 205 tumor xenografts, using pterostilbene and HPSB at a concentration of 10 mg/kg. During the experiment, all mice were monitored to investigate whether pterostilbene and HPSB treatment caused any adverse effects. As shown in **Table 2**, the body and organ weights in each group did not show any unhealthy symptoms throughout the course of the study. These results suggested that no noticeable side effects or toxicity were caused by the *i.p.* injection of pterostilbene and HPSB. Furthermore, in mice receiving these treatment regimens, no gross signs of toxicity were observed during visible inspections of general appearance and microscopic examinations of individual organs (data not shown). After15 days, tumor volume in HPSB was significantly inhibited in comparison with the pterostilbene-treated mice (**Fig. 6A and 6B**). The tumor weight was strongly inhibited in the HPSB-treated mice (**Fig. 6C**). As shown in **Figure 6D**, the protein levels of COX-2, MMP-9, VEGF, cyclin D1, and pro-caspase-3 were markedly decreased in COLO 205 xenograft tumors from the 10 mg/kg HPSB-treated group compared to the pterostilbene group. Our results suggest that HPSB could serve as a novel promising agent for cancer chemotherapeutic purposes.

**Table 2:** Effects of pterostilbene and 3′-hydroxypterostilbene administration on the body weight and organ weight in a COLO 205 xenograft model^a^.

Treatment	No. of mice	Body weight (g)	Liver (mg)	Kidney (mg)	Spleen (mg)
Control	6	19.00±1.68	0.94±0.19	0.25±0.13	0.07±0.03
Pterostilbene (10 mg/kg)	6	18.37±1.66	0.90±0.09	0.31±0.04	0.06±0.01
3′-Hydroxypterostilbene (10 mg/kg)	6	19.39±1.44	0.91±0.16	0.32±0.07	0.07±0.03

a COLO 205 cells were injected into 3–4 week old BALB/c nude mice (5×10^6^ cells per mouse). After tumors grew to about 100–200 mm^3^, mice were *i.p.* treated with pterostilbene or 3′-hydroxypterostilbene for 15 days. All mice of each group were sacrificed by CO_2_ asphyxiation at the end of experiment. Comparisons were analyzed using ANOVA followed by Fisher's least significant difference test.

## Discussion

In this study, for the first time, we compare with the cancer cell growth inhibitory effect of pterostilbene and HPSB (**Figure 1**) in human colon cancer cells. The results of this study showed that HPSB more potently induced apoptosis and autophagy than pterostilbene in COLO 205 cells. This study indicates that the difference in bioactivity of HPSB compare with pterostilbene is related to the presence and position of hydroxyl groups on the basic pterostilbene chemical structure. Human colorectal COLO 205 cancer cells underwent apoptosis following the treatment with HPSB, suggesting that apoptosis is the major cause for the growth-inhibitory effect of HPSB. This data should provide medicinal chemists with novel information for the design of anti-tumor agents, and also information to support further biological studies of these modified and unmodified functional groups in the future. As shown in **Figure 2**, HPSB is a strong inhibitor of cell viability and causes the potent and rapid induction of apoptosis in COLO 205 cells. Indeed, treatment with HPSB causes reduction of mitochondrial membrane potential and induction of caspase-3 and caspage-9, which is associated with the degradation of DFF-45 and PARP, preceded the onset of apoptosis. The role of ROS in the induction of apoptosis is well recognized. Interestingly, an increase in intracellular hydrogen peroxide levels in pterostilbene, whereas markedly decrease the hydrogen peroxide levels in HPSB-treated COLO 205 cells (**Fig. 2E**). We speculated that pterostilbene penetrates the cell membrane into cytosol and affects mitochondria cycling dioxygen through the electron transport assembly. However, HPSB could directly scavenge ROS in cells and further disrupt intracellular redox balance.

Cancer development, a dynamic and long-term process, involves many complex factors with stepwise progression ultimately leading to an uncontrolled spreading and growth of cancerous cells throughout the body called metastasis. Epidemiological studies have provided convincing evidence that dietary factors can modify this process [1]. The relationship between apoptosis and autophagy is complex and varies between cell types and different stresses. Autophagy is also genetically programmed, promoters of autophagy have the potential for clinical benefit in the setting of cancer prevention [24]. As autophagy is required for the effective management of metabolic stress, promoting autophagy through mTOR pathway inhibition might be expected to limit tumor progression [25]. Dietary natural compounds offer a great potential in the fight against human cancer by inhibiting the carcinogenesis process through cell defensive and apoptotic machineries [10]. Accumulating evidence clearly indicates that induction of apoptosis and autophagy as a mechanism of cancer prevention by dietary natural compounds [24]. We demonstrated that anti-cancer effects of HPSB are regulated by apoptosis and autophagy, and the signaling pathways mediating HPSB-induced autophagy were identified. Our results showed that after incubation with HPSB, the LC3I/II expressions was remarkably increased in COLO 205 cells (**Fig. 3D**). The PI3K/Akt and mTOR/p70S6K pathways are main signaling pathways that negatively regulate autophagy [26]. mTOR activates the downstream p70S6 Ser/Thr kinase that phosphorylates ribosomal protein S6, required for biosynthesis of the cell's translational apparatus, a critical component of cell growth and proliferation [27]. This study clearly demonstrated that PHSB inhibits the PI3K/Akt/mTOR/p70S6K and transit activates ERK1/2 and JNK1/2 pathways to promote autophagy (**Fig. 4**). To the best of our knowledge, the study is the first to demonstrate that HPSB can promote both apoptosis and autophagy in human colon cancer cells.

To the best of our knowledge, the study is the first to demonstrate that a remarkable reduction of COLO 205 xenograft tumors following oral HPSB treatment was observed (**Fig. 6**). As compared to the control, treatment with HPSB strongly induced the activation of caspase-3. Taken together, these results suggest the anti-tumor efficacy of HPSB by *i.p*. injection against colon cancer through the inhibition of inflammation, metastasis, and angiogenesis as well as through the induction of apoptosis (**Fig. 6D**). Our results strongly suggest that HPSB could be developed into a novel chemotherapeutic agent.

In summary, we have provided the basis for a molecular mechanism of HPSB in cancer treatment. The potential application of HPSB to inhibit cancer cell proliferation makes it an attractive agent for colorectal carcinoma research and possibly, treatment.
